# The influence of positive end-expiratory pressure (PEEP) in predicting fluid responsiveness in patients undergoing one-lung ventilation

**DOI:** 10.7150/ijms.59653

**Published:** 2021-04-29

**Authors:** In-Jung Jun, Mi Hwa Chung, Jung Eun Kim, Hye Sun Lee, Jung Mo Son, Eun Mi Choi

**Affiliations:** 1 Department of Anesthesiology and Pain Medicine, Kangnam Sacred Heart Hospital, University of Hallym College of Medicine, Seoul, Korea.; 2 Biostatistics Collaboration Unit, Yonsei University College of Medicine, Seoul, Korea.

**Keywords:** positive end-expiratory pressure, one-lung ventilation, pulse pressure variation, stroke volume variation, fluid responsiveness, Esophageal Doppler

## Abstract

**Background:** Dynamic preload parameters such as pulse pressure variation (PPV) and stroke volume variation (SVV) have widely been used as accurate predictors for fluid responsiveness in patients under mechanical ventilation. To circumvent the limitation of decreased cyclic change of intrathoracic pressure, we performed an intermittent PEEP challenge test to evaluate whether PPV or SVV can predict fluid responsiveness during one-lung ventilation (OLV).

**Methods:** Forty patients undergoing OLV were analyzed. Baseline hemodynamic variables including PPV and SVV and respiratory variables were recorded after chest opening in lateral position under OLV (T1). Five minutes after application of PEEP 10 cmH_2_O, the parameters were recorded (T2). Thereafter, PEEP was withdrawn to 0 cmH_2_O for 5 minutes (T3), and fluid loading was performed with balanced crystalloid solution 6 mL/kg of ideal body weight for 5 minutes. Five minutes after completion of fluid loading, all variables were recorded (T4). The patient was classified as fluid responder if SV increased ≥10% after fluid loading and as non-responder if SV increased <10%.

**Results:** Prediction of fluid responsiveness was evaluated with area under the receiver operating characteristic (ROC) curve (AUC). Change in stroke volume variation (ΔSVV) showed AUC of 0.9 (P < 0.001), 95% CI = 0.82-0.99, sensitivity = 88%, specificity = 82% for discrimination of fluid responsiveness. Change in pulse pressure variation (ΔPPV) showed AUC of 0.88 (P < 0.001), 95% CI = 0.78-0.97, sensitivity = 83%, specificity = 72% in predictability of fluid responsiveness. Cardiac index and stroke volume were well maintained after PEEP challenge in non-responders while they increased in responders.

**Conclusions:** ΔPPV and ΔSVV induced by PEEP challenge are reliable parameters to predict fluid responsiveness as well as very good predictors of fluid unresponsiveness during OLV.

## Introduction

Optimal fluid management during lung surgery is of significant importance and remains challenging for anesthesiologists. Generally, restrictive fluid management is recommended for lung surgery to prevent acute lung injury or impairment of respiratory gas exchange from fluid overload 1, 2. However, maintaining adequate organ perfusion is also important for favorable outcomes. Dynamic preload parameters such as pulse pressure variation (PPV), or stroke volume variation (SVV) have been widely used as accurate predictors for fluid responsiveness in patients under mechanical ventilation 3.

During lung surgery, fluid management using dynamic parameters is complex because of physiologic alteration by opened non-dependent thorax, one-lung ventilation (OLV) and protective ventilation technique with low tidal volume. These conditions affect the ability of PPV and SVV to predict fluid responsiveness 4. Previous studies for evaluating the usefulness of dynamic parameters in lung surgery showed diverse results 3, 5. In thoracic surgery with OLV, threshold value of dynamic parameters is usually lower than that of two lung mechanical ventilation because of decreased cyclic changes of intrathoracic pressure 6. For that reason, dynamic parameters might misread for predicting fluid responsiveness under OLV. Therefore, the intermittent tidal volume challenge or PEEP challenge can be applied to evaluate whether PPV or SVV could be a guide for fluid responsiveness by increasing cyclic changes of intrathoracic pressure. However, applying high tidal volume during OLV is associated with postoperative respiratory failure and high mortality rate 7. We chose PEEP challenge as an intermittent trial, since PEEP application could be the best way to improve intrathoracic pressure during OLV. PEEP application during OLV improves compliance and functional residual capacity of the dependent lung and improves oxygenation 8, 9.

To our knowledge, challenge test to improve the diagnostic ability of dynamic parameters have not been studied during OLV. We hypothesized that temporary increase in PEEP from 0 to 10 cmH_2_O may improve predictability of dynamic parameters on fluid responsiveness during OLV.

## Materials and Methods

### Participants

**Figure 1:**
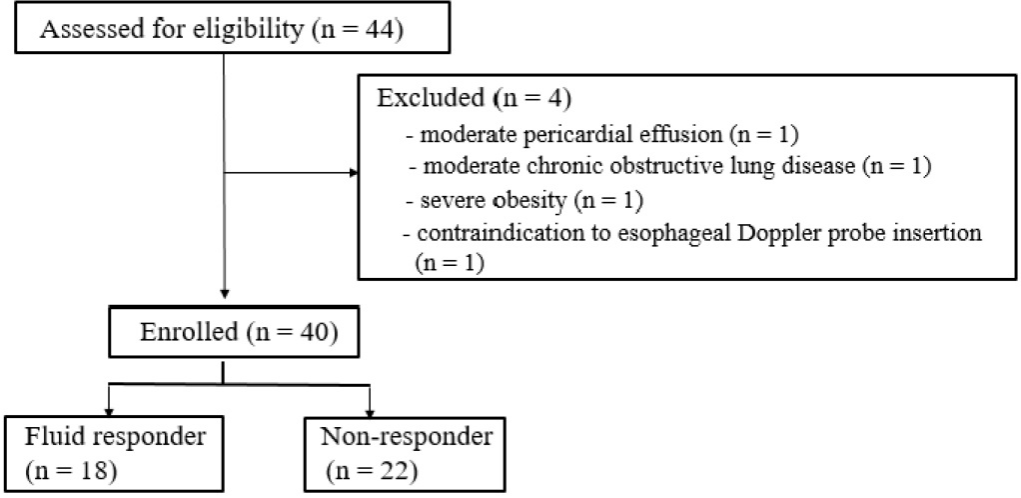
Flow diagram of the study.

After approval from the Institutional Review Board of Hallym University Kangnam Sacred Heart Hospital (approval number: 2018-11-028) and registration at ClinicalTrials.gov (NCT 03794414), we conducted the prospective observational study in 40 patients undergoing lung surgery under OLV (Fig. 1). The study was performed between January, 2019 and June, 2020 in our institution, and informed consent was obtained from all participants.

Exclusion criteria are as follows: known preoperative cardiac disease including arrhythmia, moderate to severe valvular heart disease, moderate to severe pericardial effusion, left ventricular ejection fraction <40%, moderate to severe chronic obstructive lung disease with 1-second forced expiratory volume <60% of predicted values, contraindication to esophageal Doppler monitoring probe insertion (i.e. esophageal stent, carcinoma of the esophagus or pharynx, previous esophageal surgery, esophageal stricture, esophageal varices, pharyngeal pouch and severe coagulopathy) and severe obesity (body mass index >35 kg/m^2^).

### Anesthesia and one-lung ventilation

On arrival to the operation room, routine hemodynamic monitoring including electrocardiography, pulse oximetry and non-invasive arterial pressure measurement were applied.

Induction of anesthesia was performed with continuous infusion of propofol and remifentanil using a target-controlled infusion system to induce and maintain anesthesia. Propofol was adjusted to effect site target concentration of 2-4 mcg/mL and remifentanil was adjusted to effect site target concentration of 2-8 ng/mL. Depth of anesthesia was continuously evaluated with an entropy (GE Healthcare, Datex-Ohmeda, Helsinki, Finland), and an entropy value was maintained between 40 and 60. All patients received 0.6 mg/kg of rocuronium for muscle relaxation. When maximal suppression was reached on train-of-four stimulation, left-sided or right-sided double-lumen tube (Broncho-cath, Tyco Healthcare, Argyle, Mansfield, MA, USA) was inserted for OLV. Correct position and depth was confirmed with fiberoptic bronchoscope. Radial artery was cannulated for continuous arterial blood pressure monitoring. Forced air warmer was applied and core temperature was maintained above 36.0 °C. Following induction of anesthesia, an esophageal Doppler probe (CardioQ, Deltex Medical, Chichester, UK) was inserted into the esophagus by a well-trained anesthesiologist. The esophageal Doppler probe was placed in the optimal position by monitoring aortic visual and auditory signals displaced on the CardioQ-ODM+ monitor (Deltex Medical, Chichester, UK).

Following position change to lateral position, OLV was initiated with tidal volume of 6 mL/kg of ideal body weight, an inspired oxygen fraction of 0.5 and I:E ratio of 1:1.5 without PEEP using volume-controlled mode. Respiratory rate was adjusted to maintain end-tidal carbon dioxide partial pressure between 35 and 40 mmHg. An inspired oxygen fraction was elevated by 0.1 if oxygen saturation fell below 92%.

### Hemodynamic and respiratory assessment

Heart rate, mean arterial blood pressure and PPV were recorded through the patient monitor (CARESCAPE Monitor B850; GE Healthcare). Dynamic compliance, mean airway pressure and peak inspiratory pressure were obtained from the anesthesia ventilator machine (Datex Ohmeda Avance CS Anesthesia Machine; GE Healthcare, Helsinki, Finland). Cardiac index, stroke volume and SVV were acquired from CardioQ-ODM+ monitor. PPV and SVV were calculated as follows:

PPV = [(PPmax - PPmin) / (PPmax+PPmin)/2] x 100 (%)

SVV = [(SVmax - SVmin) / (SVmax+SVmin)/2] x 100 (%)

The parameters were displayed on the CardioQ-ODM+ monitor every 10 seconds. The parameters were recorded on three consecutive respiratory cycles and averaged for statistical analysis. Each measurements were performed under stable hemodynamic state with no inotropes or vasopressors. The changes in PPV and SVV after PEEP challenge were calculated as follows:

ΔPPV = PPV_2_ (PPV measured at T2) - PPV_1_ (PPV measured at T1)

ΔSVV = SVV_2_ (SVV measured at T2) - SVV_1_ (SVV measured at T1)

### Study design

**Figure 2:**
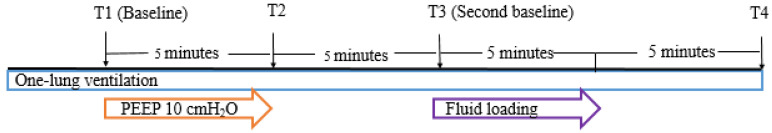
Study protocol. T1: baseline measurement of hemodynamic and respiratory parameters 20 minutes after one-lung ventilation; T2: 5 minutes after application of PEEP 10 cmH_2_O; T3: second baseline measurement 5 minutes after withdrawal of PEEP; T4: 5 minutes after completion of fluid loading. Abbreviation: PEEP: positive end-expiratory pressure.

The hemodynamic and respiratory parameters were recorded at 4 predetermined time points (Fig. 2). Baseline hemodynamic and respiratory parameters were recorded 20 minutes after initiation of the operation with OLV in lateral position (T1). After T1 measurement, 10 cmH_2_O of PEEP was applied. Five minutes after application of PEEP 10 cmH_2_O, the parameters were recorded (T2). Thereafter, PEEP was withdrawn to 0 cmH_2_O for 5 minutes and the parameters were recorded (T3). Fluid loading was performed with balanced crystalloid solution (Plasma solution A, CJ HealthCare, Seoul, South Korea) 6 mL/kg of ideal body weight for 5 minutes. Five minutes after completion of fluid loading, all parameters were recorded (T4). All measurements were made in a hemodynamically stable state with minimum or no surgical manipulation.

To determine fluid responders, stroke volume displayed on the CardioQ-ODM+ monitor was analyzed. The patient was classified as fluid responder if stroke volume increased ≥10% after fluid loading and as non-responder if stroke volume increased <10% 10.

### Statistical analysis

Statistical analysis was performed using Medcalc^®^ Version 10.0.1.0. and SPSS 22 for Windows (SPSS Inc., Chicago, IL). All data are expressed as mean ± SD, median (interquartile range) or number (percentage). Comparisons of demographic data between responders and non-responders were assessed with Student's t-test for continuous variables and Chi-square test for categorical variables. For nonparametric data analysis, Mann-Whitney U test or Fisher's exact test were used. Comparisons of hemodynamic variables before and after PEEP challenge or fluid loading were assessed with paired t-test. For nonparametric data analysis, Wilcoxon signed rank test was used.

ROC curves were generated for PPV1, PPV2, △PPV, SVV1, SVV2 and △SVV. To determine the diagnostic ability of the variables to predict fluid responsiveness, the area under the curve (AUC = 0.5: no apparent distributional difference between the two groups, no prediction possible; 0.6≤AUC<0.7: poor discrimination; 0.7≤AUC<0.8: acceptable discrimination; 0.8≤AUC<0.9: excellent discrimination; 0.9≤AUC<1.0: outstanding discrimination; AUC=1.0: best possible prediction) were calculated and compared using Hanley-McNeil test 11, 12. The optimal cut-off value was determined by considering the values that maximized sensitivity and specificity. In all analysis, P<0.05 was considered to be statically significant.

A gray zone was used to determine the values which provide inconclusive information using Youden's index. The values in the range of gray zone mean formal conclusion cannot be obtained. Inconclusive region was defined as values with a sensitivity lower than 90% or specificity lower than 90% 13.

### Sample size estimation

The sample size was calculated using PASS (version 12, NCSS, LLC, Kaysville, Utah, USA). The sample size was determined on the basis of ROC analysis. We considered augmented PPV by PEEP challenge to predict fluid responsiveness if the area under the ROC curve was >0.8. Assuming that the null hypothesis was 0.5 (no discrimination) with type 1 error = 0.05 and type 2 error = 0.1, a minimum of 36 patients were needed. Considering a dropout rate of 10%, 40 patients were enrolled.

## Results

### Patient characteristics

In our study, 44 patients were assessed for recruitment. Four patients met our exclusion criteria (moderate pericardial effusion, moderate chronic obstructive lung disease, severe obesity, contraindication of esophageal Doppler probe insertion), and 40 patients were analyzed (Fig. 1). 18 patients of 40 patients were responders to fluid loading and 22 patients were non-responders. There were no significant differences in the demographic and perioperative data between the responders and the non-responders except operation time and anesthesia time (Table 1).

### Hemodynamic and respiratory variables before and after PEEP challenge

Hemodynamic and respiratory variables at baseline (T1) and after PEEP challenge (T2) are described in Table 2. PPV and SVV increased after PEEP challenge in responders (P<0.001). Dynamic compliance, mean airway pressure and peak inspiratory pressure increased significantly in responders and non-responders after PEEP challenge. Cardiac index and stroke volume were well maintained after PEEP challenge compared to the baseline in non-responders while they increased in responders.

### Hemodynamic and respiratory variables before and after fluid loading

Hemodynamic and respiratory variables before (T3) and after fluid loading (T4) are described in Table 3. PPV and SVV decreased after fluid loading in responders. Hemodynamic variables including cardiac index and stroke volume increased significantly after fluid loading in responders. There were no significant difference in heart rate and mean arterial pressure in both responders and non-responders.

### ROC analysis

**Figure 3:**
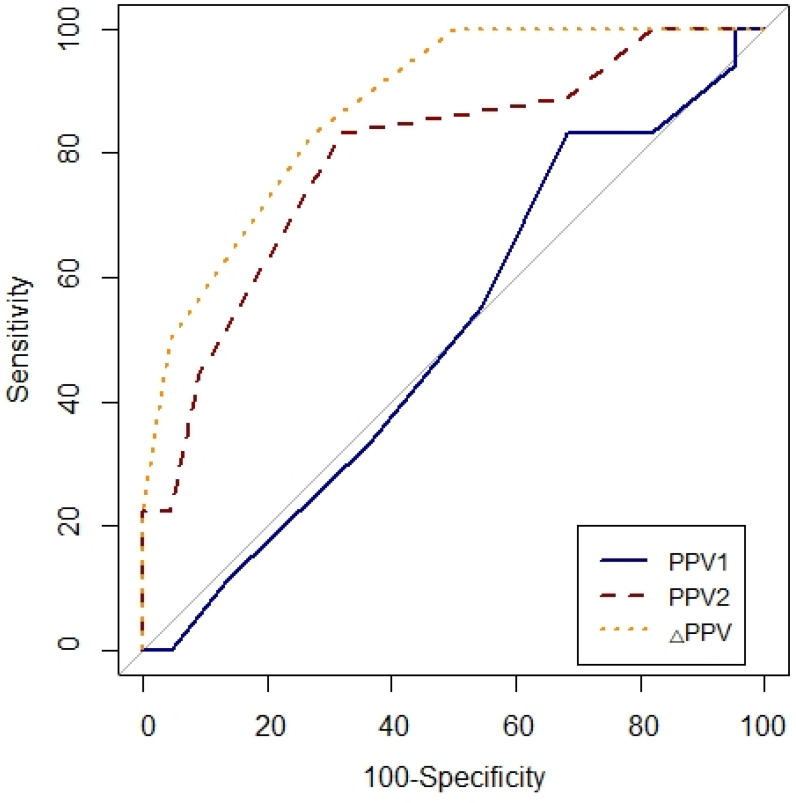
Comparison of receiver-operating characteristic curves of PPV_1_, PPV_2_ and ΔPPV to predict fluid responsiveness during one-lung ventilation. Abbreviation: PPV_1_, pulse pressure variation at baseline (T1) without PEEP; PPV_2_, pulse pressure variation at T2 with PEEP 10 cmH_2_O; ΔPPV, change in pulse pressure variation after PEEP challenge.

**Figure 4:**
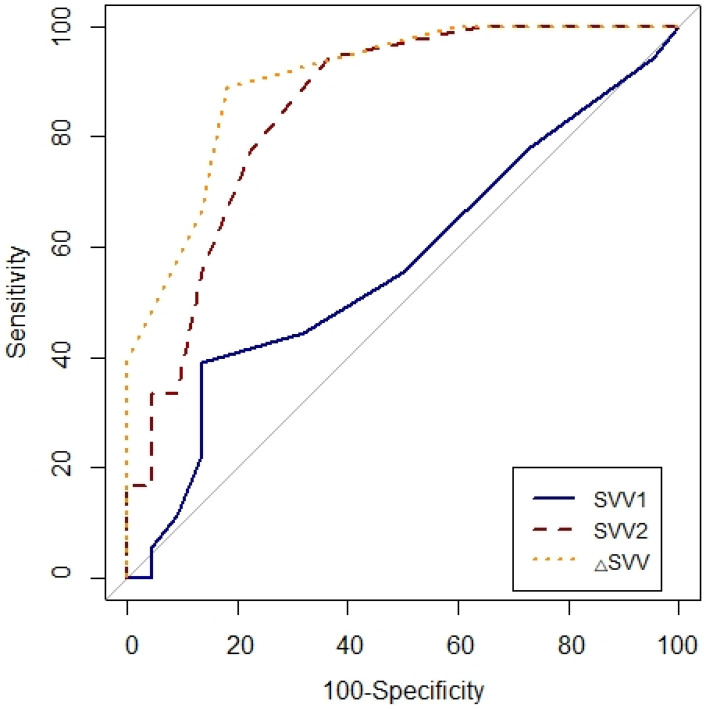
Comparison of receiver-operating characteristic curves of SVV_1_, SVV_2_ and △SVV to predict fluid responsiveness during one-lung ventilation. SVV_1_, stroke volume variation at baseline (T1) without PEEP; SVV_2_, stroke volume at T2 with PEEP 10 cmH_2_O; △SVV, change in stroke volume variation after PEEP challenge.

Prediction of fluid responsiveness was evaluated using ROC analysis (Table 4, Figs. 3 and 4). The threshold of PPV_1_, PPV_2_, ΔPPV, SVV_1_, SVV_2_ and ΔSVV were used to determine responders and non-responders with ROC analysis. ΔSVV showed successful discrimination of fluid responsiveness (AUC = 0.9, 95% CI = 0.82-0.99, P < 0.001, cut-off value > 0%, sensitivity = 88%, specificity = 82%, (+) predictive value = 80, 95% CI = 62-97, (-) predictive value = 90, 95% CI = 76-100). ΔPPV showed AUC of 0.88 (95% CI = 0.78-0.97, P < 0.001, cut-off value > 0%, sensitivity = 83%, specificity = 72%, (+) predictive value = 71, 95% CI = 52-90, (-) predictive value = 84, 95% CI = 67-100) and SVV_2_ showed AUC of 0.85 (95% CI = 0.73-0.97, P < 0.001, cut-off value > 4%, sensitivity = 94%, specificity = 63%) in predicting fluid responsiveness. Also, ΔSVV and ΔPPV were very good predictors of fluid unresponsiveness during OLV. AUC of PPV_2_ in predicting fluid responsiveness was 0.79 (P < 0.001). However, PPV_1_ and SVV_1_ did not predict fluid responsiveness.

### Gray zone analysis

**Figure 5:**
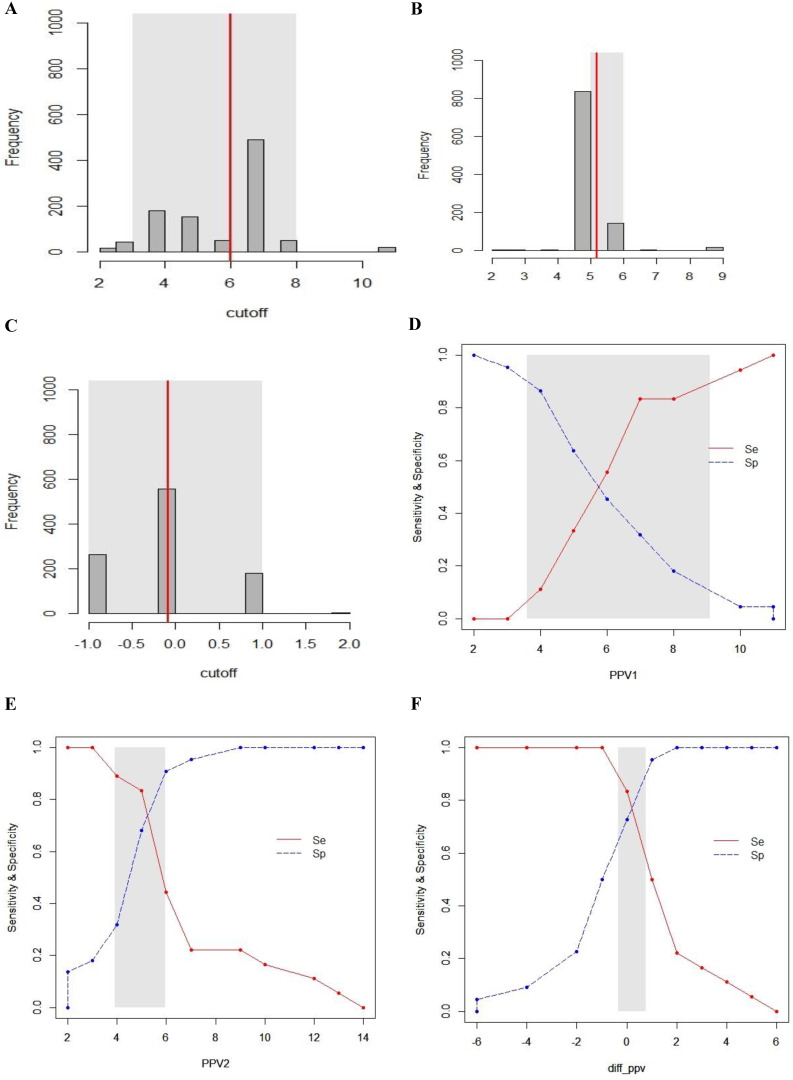
Gray zone analysis of PPV_1_, PPV_2_ and ΔPPV. The gray zone analysis depicted two cut-offs between which the diagnosis of fluid responsiveness was uncertain. The optimal threshold of 1,000 resampled population is shown by histograms and the gray zone (95% CI for the optimal cut-off) is represented as a gray shaded area (A, B and C). Using the alternative approach of splitting the ROC curve, the two curves (Se; sensitivity, red, solid line), (Sp; specificity, blue, dotted line) were retrieved (D, E and F). The inconclusive zone, which is more than 10% of diagnosis tolerance, is represented as a gray shaded area.

**Figure 6:**
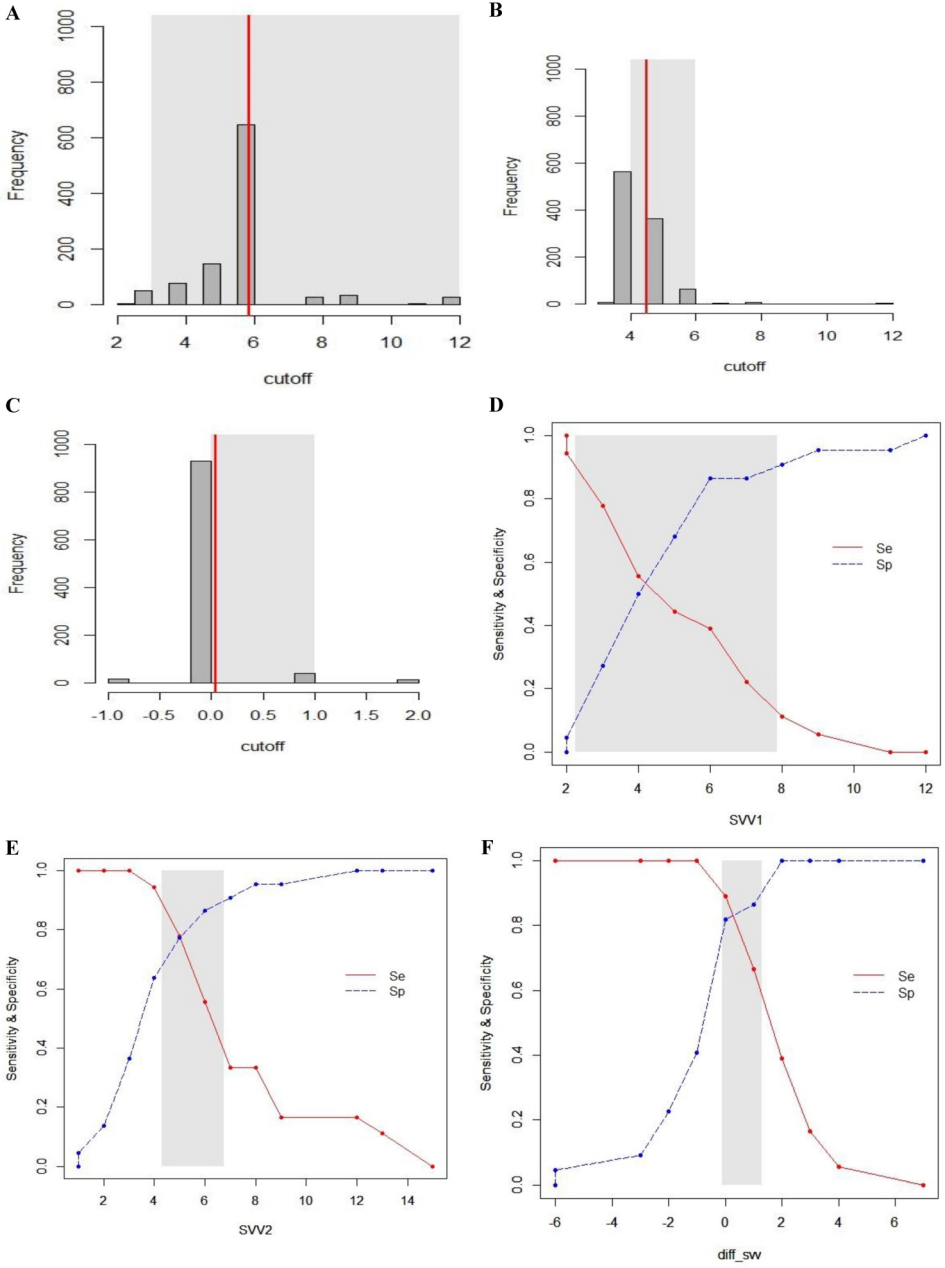
Gray zone analysis of SVV_1_, SVV_2_ and ΔSVV. The gray zone analysis depicted two cut-offs between which the diagnosis of fluid responsiveness was uncertain. The optimal threshold of 1,000 resampled population is shown by histograms and the gray zone (95% CI for the optimal cut-off) is represented as a gray shaded area (A, B and C). Using the alternative approach of splitting the ROC curve, the two curves (Se; sensitivity, red, solid line), (Sp; specificity, blue, dotted line) were retrieved (D, E and F). The inconclusive zone, which is more than 10% of diagnosis tolerance, is represented as a gray shaded area.

The inconclusive region of dynamic parameters (PPV_1_, PPV_2_, ΔPPV, SVV_1_, SVV_2_, ΔSVV) to predict fluid responsiveness was depicted using gray zone analysis (Figs. 5 and 6). By determining the resampled populations, optimal threshold was obtained (Fig. 5A, B, C), (Fig. 6A, B, C). Using the alternative approach of splitting the ROC curve into the curves with sensitivity and specificity, an inconclusive zone was retrieved (Fig. 5D, E, F), (Fig. 6D, E, F). The gray zone of PPV_1_ was between 3.6 and 9.1 with 33 numbers of patients. However, by PEEP application, the gray zone of PPV_2_ decreased from 3.9 to 5.9 with 14 numbers of patients. ΔPPV showed narrowest gray zone (-0.35 to 0.75) and only 8 patients were included. The gray zone of SVV_1_ was between 2.3 and 7.9 with 31 numbers of patients. By PEEP application, the gray zone of SVV_2_ also decreased from 4.3 to 6.8 with 12 numbers of patients. ΔSVV showed gray zone from -0.1 to 1.3 and 16 patients were included.

## Discussion

In our study, PEEP challenge maneuver from 0 to 10 cmH_2_O increased the diagnostic ability of PPV and SVV of predicting fluid responsiveness during OLV. Particularly, ΔPPV and ΔSVV induced by PEEP application were accurate predictors of fluid responsiveness.

Previous studies have noted the usefulness of PPV and SVV as predictors of fluid responsiveness during mechanical ventilation 14-16. This is based on the heart-lung interaction during positive pressure mechanical ventilation. Positive pressure derived from mechanical ventilation induces cyclic changes in the right and left heart volume loads 17. The wide changes in stroke volume during mechanical ventilation means the patient is likely to be fluid responsive 18.

In thoracic surgery, a difference in dynamic indices between responders and non-responders may be small or erratic by several reasons. During lung surgeries under OLV, the non-ventilated lung does not generate cyclic changes in intrathoracic pressure and the operating side of the chest is opened. Hence, wide range of the pressure generated from mechanical ventilation is transmitted to the atmosphere 19. Additionally, the patient develops 20% to 30% of intrapulmonary shunt in the non-dependent lung after hypoxic pulmonary vasoconstriction 20. Shunt does not take part in cyclic changes of stroke volume and the amount of pulmonary shunt left in the non-dependent lung would decrease the value of dynamic indices. For another reason, protective ventilation with small tidal volume under OLV produces reduced variations in pleural and trans-pulmonary pressure which decrease the cyclic changes in dynamic indices 6, 19. For these reasons, PPV threshold to predict fluid responsiveness is reported to be lower during OLV 3.

To overcome the several limited conditions which make dynamic parameters unreliable as predictors of fluid responsiveness, tidal volume challenge is widely used. Transient increase of tidal volume from 6 to 8 mL/kg for 1 minute increases PPV value by more than 3.5% which reliably increases the predictive ability of PPV on fluid responsiveness 21. Since applying high tidal volume may be harmful during OLV, we performed PEEP challenge by increasing intrathoracic pressure of the dependent lung. PEEP application increases pleural pressure and trans-pulmonary pressure which decreases right ventricular (RV) preload and increases RV afterload. During OLV, pleural pressure of closed dependent pleura may still take part in cyclic changes of intrathoracic pressure and affect RV preload. Consequently, higher level of PEEP results in greater cyclic changes in stroke volume which causes higher PPV and SVV 22. In the present study, although baseline PPV and SVV were low, the values increased after PEEP application in fluid responders. Similarly, Lee et al. reported that PPV in group of protective OLV with PEEP of 5 cmH_2_O can predict fluid responsiveness while PPV failed to predict fluid responsiveness during conventional OLV without PEEP 3. Fu et al. also reported that PPV and SVV can predict fluid responsiveness during protective OLV with PEEP of 5 cmH_2_O compared to conventional OLV without PEEP 19. The authors showed the role of PEEP to increase the intrathoracic pressure as an explanation. Though, there is a difference that they used PEEP as an initial ventilator setting and we applied PEEP as an intermittent trial.

The present study has the clinical implication that the simple PEEP application for short period of time improved the predictive power of PPV and SVV of fluid responsiveness in patients within the gray zone. Also, there was no decrease in cardiac index and stroke volume with optimal level of PEEP application. Rather, cardiac index and stroke volume increased only in fluid responders after PEEP application. It is not clear but we assume it is associated with hypoxic pulmonary vasoconstriction since PEEP (5 to 10 cmH_2_O) application to the dependent lung is known to help reduce blood flow to the nondependent lung. High level of PEEP (15-20 cmH_2_O) is known to decrease cardiac output, although the precise mechanism to PEEP during OLV is not simple and the intrathoracic response appear multiple and complex 23.

There are several limitations with the present study. First, the total number of patients may not be sufficient to generalize our result to all patients. Further studies with larger number of patients calculated with precise parameter are needed to describe the physiologic mechanism of PEEP on dynamic parameters during OLV. Second, we used esophageal Doppler for measurement of dynamic parameters. Esophageal Doppler probe frequently needed repositioning during the operation. However, we measured cardiac output when optimal signal from descending aorta was obtained with minimum surgical manipulation. Several studies have evaluated the use of esophageal Doppler during OLV and validated its use of measuring cardiac output compared to standard method of thermodilution 24-26. Third, operation time and anesthesia time were statistically significantly different between the responders and non-responders. Since parameters were measured at the beginning of the operation, operation time is irrelevant with the result. Fourth, two types of surgeries (video-assisted thoracic surgery, open thoracotomy) were included for analysis. Although only two patients underwent open thoracotomy, further study including either one type of surgery may be warranted.

In conclusion, the present study demonstrates that PEEP challenge maneuver increased the diagnostic ability of PPV and SVV of predicting fluid responsiveness and fluid unresponsiveness during OLV. The use of ΔPPV and ΔSVV after PEEP challenge would be helpful to guide fluid management in patients under OLV.

## Figures and Tables

**Table 1:** Demographic and perioperative data

Variables*	Responders (n = 18)	Non-responders (n = 22)	*P*-value
Age (years)	48 ± 20.9	57.8 ± 16.8	0.113
Gender (male/female)	16/2	14/8	0.082
Height (cm)	168.6 ± 7.6	162.9 ± 11.2	0.077
Weight (kg)	61.2 ± 8.6	64.9 ± 12.1	0.264
Ideal body weight (kg)	63.4 ± 7.5	57.8 ± 12.3	0.098
Body mass index (kg/m^2^)	22.1 ± 2.4	23.0 ± 3.7	0.362
Hypertension	4 (22)	6 (27)	0.503
Operation time (min)	123.06 ± 71.6	209.8 ± 94.3	0.002
Anesthesia time (min)	173.9 ± 82.9	264.1 ± 101.4	0.004
Intraoperative fluid amount (mL)	1061.1 ± 426	1297.1 ± 535.9	0.129
Operation type (Open thoracotomy/video-assisted thoracic surgery)	0/18	2/20	0.492
Side of the operation (Right/Left)	13/5	14/8	0.564

*Data are shown as mean ± standard deviation or number (percentage).

**Table 2:** Hemodynamic and respiratory variables at baseline and after PEEP challenge

Variables*	Baseline (T1)	PEEP challenge (T2)	Change	*P*-value
**Heart rate (beats/min)**				
Responders	78.7 ± 15.8	81.1 ± 15.5	2.4 ± 5.5	0.085
Non-responders	72.6 ± 9.9	70.6 ± 8.2	-1.9 ± 3.6	0.018
**Mean arterial pressure (mmHg)**				
Responders	91.9 ± 15.3	84.6 ± 17.0	-7.3 ± 8.5	0.002
Non-responders	89.1 ± 12.6	88.0 ± 13.9	-1.1 ± 6.1	0.414
**Heart rate/respiratory rate ratio**				
Responders	6.2 ± 1.1	6.3 ± 1.2	0.2 ± 0.4	0.075
Non-responders	5.8 ± 0.9	5.6 ± 0.8	-0.2 ± 0.3	0.020
**Dynamic compliance**				
Responders	23.8 ± 6.1	26.9 ± 7.9	3.1 ± 5.4	0.025
Non-responders	21.5 ± 5.1	25.6 ± 4.8	4.1 ± 2.7	<0.001
**Mean airway pressure (cmH_2_O)**				
Responders	7 (7-8)	14 (13-14.3)	7 (6-7.3)	<0.001
Non-responders	7 (6-8)	14.5 (14-15)	7.5 (6.7-8)	<0.001
**Peak inspiratory pressure (cmH_2_O)**				
Responders	18.2 ± 3.5	23.2 ± 2.7	5.1 ± 1.3	<0.001
Non-responders	18.6 ± 3.5	23.8 ± 2.3	5.2 ± 2.0	<0.001
**Cardiac index (L/min/m^2^)**				
Responders	2.7 ± 0.6	2.9 ± 0.7	0.3 ± 0.4	0.013
Non-responders	3 ± 1.0	3 ± 0.9	-0.04 ± 0.3	0.624
**Stroke volume (mL)**				
Responders	58.3 ± 12.9	63.7 ± 16.8	5.4 ± 7.3	0.006
Non-responders	71.5 ± 19.3	71.5 ± 19.7	0.0 ± 4.4	1.000
**Pulse pressure variation (%)**				
Responders	5.4 ± 1.8	7.3 ± 2.9	1.9 ± 1.7	<0.001
Non-responders	5.5 ± 2.1	4.8 ± 1.7	-0.7 ± 1.8	0.092
**Stroke volume variation (%)**				
Responders	5.5 ± 2.4	7.8 ± 3.3	2.3 ± 1.7	<0.001
Non-responders	5.0 ± 2.3	4.4 ± 2.4	-0.5 ± 1.8	0.168

Abbreviation: PEEP: positive end-expiratory pressure. *Data are shown as mean ± standard deviation or median (interquartile range).

**Table 3:** Hemodynamic and respiratory variables before and after fluid loading

Variables*	Second baseline (T3)	After fluid loading (T4)	Change	*P*-value
**Heart rate (beats/min)**				
Responders	79.2 ± 15.5	79.4 ± 14.6	0.2 ± 6.9	0.920
Non-responders	69.3 ± 8.8	68.3 ± 9.7	-1.0 ± 4.1	0.269
**Mean arterial pressure (mmHg)**				
Responders	84.4 ± 13.4	83.1 ± 13.5	-1.3 ± 7.4	0.452
Non-responders	87.8 ± 14.0	88.7 ± 10.6	0.9 ± 6.9	0.566
**Heart rate/respiratory rate ratio**				
Responders	6.2 ± 1.3	6.2 ± 1.1	-0.0 ± 0.5	0.913
Non-responders	5.5 ± 0.8	5.4 ± 0.8	-0.1 ± 0.3	0.263
**Dynamic compliance**				
Responders	23.9 ± 7.7	23.0 ± 6.0	-0.9 ± 3.9	0.354
Non-responders	22.3 ± 4.5	21.0 ± 4.2	-1.3 ± 1.5	0.001
**Mean airway pressure (cmH_2_O)**				
Responders	7.1 ± 1.2	7.3 ± 1.4	0.2 ± 0.4	0.042
Non-responders	7.1 ± 1.1	7.2 ± 1.1	0 (0-0)	0.162
**Peak inspiratory pressure (cmH_2_O)**				
Responders	17.4 ± 3.2	18.5 ± 3.5	1.1 ± 0.9	<0.001
Non-responders	17.9 ± 3.4	18.8 ± 3.3	0.9 ± 0.9	<0.001
**Cardiac index (L/min/m^2^)**				
Responders	2.9 ± 0.7	3.4 ± 0.9	0.5 ± 0.7	0.006
Non-responders	2.9 ± 0.9	3.1 ± 0.8	0.1 ± 0.3	0.138
**Stroke volume (mL)**				
Responders	63.1 ± 17.2	77.2 ± 16.8	14.1 ± 8.5	<0.001
Non-responders	72.3 ± 17.7	74.3 ± 17.5	1.9 ± 6.4	0.166
**Pulse pressure variation (%)**				
Responders	5.8 ± 2.4	4.2 ± 2.1	-1.6 ± 1.2	<0.001
Non-responders	5.6 ± 1.8	5 ± 1.4	-0.6 ± 1.4	0.056
**Stroke volume variation (%)**				
Responders	5.7 ± 2.4	4.2 ± 1.9	-1.6 ± 1.2	<0.001
Non-responders	5.2 ± 2.5	4.7 ± 2.3	-0.5 ± 1.7	0.143

*Data are shown as mean ± standard deviation.

**Table 4:** Prediction of fluid responsiveness by the ROC curves of variables measured before or after fluid loading

	AUC (95% CI)	*P*-value	Cut-off value, %	Sensitivity (95% CI)	Specificity (95% CI)	(+) predictive value (95% CI)	(-) predictive value (95% CI)
PPV_1_	0.51 (0.33-0.69)	0.902	6	83 (66-100)	31 (12-51)	50 (32-67)	70 (41-98)
PPV_2_	0.79 (0.66-0.93)	<0.001	5	83 (66-100)	68 (48-87)	68 (48-87)	83 (66-100)
ΔPPV	0.88 (0.78-0.97)	<0.001	0	83 (66-100)	72 (54-91)	71 (52-90)	84 (67-100)
SVV_1_	0.57 (0.39-0.75)	0.459	6	38 (16-61)	86 (72-100)	70 (41-98)	63 (46-80)
SVV_2_	0.85 (0.73-0.97)	<0.001	4	94 (83-100)	63 (43-83)	68 (49-86)	93 (80-100)
ΔSVV	0.9 (0.82-0.99)	<0.001	0	88 (74-100)	82 (65-97)	80 (62-97)	90 (76-100)

Abbreviation: ROC: receiver operating characteristic, AUC: area under the curve, CI: confidence interval, PPV_1_: pulse pressure variation at baseline (T1), PPV_2_: pulse pressure variation during PEEP (positive end-expiratory pressure) challenge, ΔPPV: change in pulse pressure variation after PEEP challenge, SVV_1_: stroke volume variation at baseline (T1), SVV_2_: stroke volume variation during PEEP challenge, ΔSVV: change in stroke volume variation after PEEP challenge.

## References

[1] Zhang J, Chen CQ, Lei XZ, Feng ZY, Zhu SM (2013). Goal-directed fluid optimization based on stroke volume variation and cardiac index during one-lung ventilation in patients undergoing thoracoscopy lobectomy operations: a pilot study. Clinics (Sao Paulo).

[2] Chau EH, Slinger P (2014). Perioperative fluid management for pulmonary resection surgery and esophagectomy. Semin Cardiothorac Vasc Anesth.

[3] Lee J-H, Jeon Y, Bahk J-H, Gil N-S, Hong DM, Kim JH (2011). Pulse pressure variation as a predictor of fluid responsiveness during one-lung ventilation for lung surgery using thoracotomy: randomised controlled study. European Journal of Anaesthesiology (EJA).

[4] Suehiro K, Okutani R (2011). Influence of tidal volume for stroke volume variation to predict fluid responsiveness in patients undergoing one-lung ventilation. J Anesth.

[5] Jeong DM, Ahn HJ, Park HW, Yang M, Kim J, Park J (2017). Stroke volume variation and pulse pressure variation are not useful for predicting fluid responsiveness in thoracic surgery. Anesthesia & Analgesia.

[6] Batchelor TJP, Rasburn NJ, Abdelnour-Berchtold E, Brunelli A, Cerfolio RJ, Gonzalez M (2019). Guidelines for enhanced recovery after lung surgery: recommendations of the Enhanced Recovery After Surgery (ERAS(R)) Society and the European Society of Thoracic Surgeons (ESTS). Eur J Cardiothorac Surg.

[7] Trepte CJ, Haas SA, Nitzschke R, Salzwedel C, Goetz AE, Reuter DA (2013). Prediction of volume-responsiveness during one-lung ventilation: a comparison of static, volumetric, and dynamic parameters of cardiac preload. J Cardiothorac Vasc Anesth.

[8] Kang WS, Kim SH, Kim SY, Oh CS, Lee SA, Kim JS (2014). The influence of positive end-expiratory pressure on stroke volume variation in patients undergoing cardiac surgery: an observational study. J Thorac Cardiovasc Surg.

[9] Senturk M, Slinger P, Cohen E (2015). Intraoperative mechanical ventilation strategies for one-lung ventilation. Best Pract Res Clin Anaesthesiol.

[10] Carsetti A, Cecconi M, Rhodes A (2015). Fluid bolus therapy: monitoring and predicting fluid responsiveness. Curr Opin Crit Care.

[11] Hanley JA, McNeil BJ (1983). A method of comparing the areas under receiver operating characteristic curves derived from the same cases. Radiology.

[12] Unal I (2017). Defining an Optimal Cut-Point Value in ROC Analysis: An Alternative Approach. Comput Math Methods Med.

[13] Cannesson M, Le Manach Y, Hofer CK, Goarin JP, Lehot JJ, Vallet B (2011). Assessing the diagnostic accuracy of pulse pressure variations for the prediction of fluid responsiveness: a "gray zone" approach. Anesthesiology.

[14] Cannesson M, Musard H, Desebbe O, Boucau C, Simon R, Henaine R (2009). The ability of stroke volume variations obtained with Vigileo/FloTrac system to monitor fluid responsiveness in mechanically ventilated patients. Anesth Analg.

[15] Bendjelid K, Romand JA (2003). Fluid responsiveness in mechanically ventilated patients: a review of indices used in intensive care. Intensive Care Med.

[16] Min JJ, Kim TK, Lee JH, Park J, Cho HS, Kim WS (2017). Evaluation of augmented pulse pressure variation using the Valsalva manoeuvre as a predictor of fluid responsiveness under open-chest conditions: A prospective observational study. Eur J Anaesthesiol.

[17] Jozwiak M, Monnet X, Teboul JL (2018). Prediction of fluid responsiveness in ventilated patients. Ann Transl Med.

[18] Michard F, Teboul J-L (2000). Using heart-lung interactions to assess fluid responsiveness during mechanical ventilation. Critical Care.

[19] Fu Q, Duan M, Zhao F, Mi W (2015). Evaluation of stroke volume variation and pulse pressure variation as predictors of fluid responsiveness in patients undergoing protective one-lung ventilation. Drug Discov Ther.

[20] Beck DH, Doepfmer UR, Sinemus C, Bloch A, Schenk MR, Kox WJ (2001). Effects of sevoflurane and propofol on pulmonary shunt fraction during one-lung ventilation for thoracic surgery. Br J Anaesth.

[21] Myatra SN, Prabu NR, Divatia JV, Monnet X, Kulkarni AP, Teboul JL (2017). The Changes in Pulse Pressure Variation or Stroke Volume Variation after a “Tidal Volume Challenge” Reliably Predict Fluid Responsiveness During Low Tidal Volume Ventilation. Crit Care Med.

[22] Kubitz JC, Annecke T, Kemming GI, Forkl S, Kronas N, Goetz AE (2006). The influence of positive end-expiratory pressure on stroke volume variation and central blood volume during open and closed chest conditions. Eur J Cardiothorac Surg.

[23] Luecke T, Pelosi P (2005). Clinical review: Positive end-expiratory pressure and cardiac output. Crit Care.

[24] Diaper J, Ellenberger C, Villiger Y, Robert J, Inan C, Tschopp J-M (2010). Comparison of cardiac output as assessed by transesophageal echo-Doppler and transpulmonary thermodilution in patients undergoing thoracic surgery. Journal of clinical anesthesia.

[25] Diaper J, Ellenberger C, Villiger Y, Robert J, Tschopp J-M, Licker M (2008). Transoesophageal Doppler monitoring for fluid and hemodynamic treatment during lung surgery. Journal of clinical monitoring and computing.

[26] Kaufmann KB, Stein L, Bogatyreva L, Ulbrich F, Kaifi JT, Hauschke D (2017). Oesophageal Doppler guided goal-directed haemodynamic therapy in thoracic surgery - a single centre randomized parallel-arm trial. Br J Anaesth.

